# Deep learning as a novel method for endoscopic diagnosis of chronic atrophic gastritis: a prospective nested case–control study

**DOI:** 10.1186/s12876-022-02427-2

**Published:** 2022-07-25

**Authors:** Quchuan Zhao, Qing Jia, Tianyu Chi

**Affiliations:** 1 Department of Gastroenterology, Xuanwu Hospital of Capital Medical University, 45 Chang-chun Street, Beijing, 100053 China; 2 Department of Anesthesiology, Guang’anmen Hospital China Academy of Chinese Medical Sciences, 5 North Court Street, Beijing, 100053 China

**Keywords:** Artificial intelligence, Deep learning, U-Net, Gastroscopy, Chronic atrophic gastritis

## Abstract

**Background and aims:**

Chronic atrophic gastritis (CAG) is a precancerous disease that often leads to the development of gastric cancer (GC) and is positively correlated with GC morbidity. However, the sensitivity of the endoscopic diagnosis of CAG is only 42%. Therefore, we developed a real-time video monitoring model for endoscopic diagnosis of CAG based on U-Net deep learning (DL) and conducted a prospective nested case–control study to evaluate the diagnostic evaluation indices of the model and its consistency with pathological diagnosis.

**Methods:**

Our cohort consisted of 1539 patients undergoing gastroscopy from December 1, 2020, to July 1, 2021. Based on pathological diagnosis, patients in the cohort were divided into the CAG group or the chronic nonatrophic gastritis (CNAG) group, and we assessed the diagnostic evaluation indices of this model and its consistency with pathological diagnosis after propensity score matching (PSM) to minimize selection bias in the study.

**Results:**

After matching, the diagnostic evaluation indices and consistency evaluation of the model were better than those of endoscopists [sensitivity (84.02% vs. 62.72%), specificity (97.04% vs. 81.95%), positive predictive value (96.60% vs. 77.66%), negative predictive value (85.86% vs. 68.73%), accuracy rate (90.53% vs. 72.34%), Youden index (81.06% vs. 44.67%), odd product (172.5 vs. 7.64), positive likelihood ratio (28.39 vs. 3.47), negative likelihood ratio (0.16 vs. 0.45), AUC (95% CI) [0.909 (0.884–0.934) vs. 0.740 (0.702–0.778)] and Kappa (0.852 vs. 0.558)].

**Conclusions:**

Our prospective nested case–control study proved that the diagnostic evaluation indices and consistency evaluation of the real-time video monitoring model for endoscopic diagnosis of CAG based on U-Net DL were superior to those of endoscopists.

*Trial registration* ChiCTR2100044458, 18/03/2020.

## Introduction

China is a country with a high gastric cancer (GC) morbidity. Every year, there are approximately 400,000 new cases of GC and approximately 350,000 deaths. The number of new cases and deaths accounts for 40% of the world's GC cases. The prognosis of GC is closely related to the timing of diagnosis and treatment. Early detection, early diagnosis and early treatment of cancer have always been the main strategies to reduce mortality and improve survival [1]. Chronic atrophic gastritis (CAG) is a precancerous disease of GC that positively correlates with GC morbidity [2, 3]. Early diagnosis and treatment of CAG is an efficient and feasible way to modify the severe situation of diagnosis and treatment of GC. However, if pathological diagnosis is taken as the "gold standard", the sensitivity of the endoscopic diagnosis of CAG is only 42% [4]. Therefore, determining how to improve the diagnostic rate of CAG and the coincidence rate between endoscopy and pathological diagnosis has been a hot issue of clinical attention.

In recent years, artificial intelligence (AI) has made breakthroughs in the field of image recognition. In particular, the emergence of deep learning (DL) has eliminated the need for the artificial extraction of data features, which is inefficient and incomplete [5–7]. Although the DL technique combined with digestive endoscopy has become one of the hot topics in the field of digestive research [8–10], research on the application of DL in the identification of CAG remains less common; most of the research on training and validation of the model has used static images from retrospective data, and less of the research has been on the identification of real-time video monitoring [11–13].

Therefore, we developed a real-time video monitoring model for endoscopic diagnosis of CAG based on U-Net DL. Our team previously proved through a prospective cohort study that the real-time video monitoring model for endoscopic diagnosis of CAG based on U-Net DL can improve the endoscopic diagnosis rate of CAG compared with that of endoscopists [14]. To further verify the performance of the model, we enrolled additional patients into the cohort and conducted a prospective nested case–control study to evaluate the diagnostic evaluation indices of the model and its consistency with pathological diagnosis, using pathological diagnosis as the gold standard.

## Methods

### Sample size calculation

PASS 15 (NCSS, LCC., Kaysville, Utah) was used to calculate the sample size. We planned to use patients in the cohort to conduct a prospective nested case–control study to verify the sensitivity, specificity and other diagnostic evaluation indices of the DL model for CAG. The operational process was as follows: Proportions → One Proportion → Confidence Interval → Confidence Interval for One Proportion. According to the guidelines, with pathological diagnosis as the "gold standard", the sensitivity and specificity of endoscopic diagnosis of atrophy are only 42% and 91% [4], respectively. We assumed that the DL model could improve the sensitivity by 50%, and we set α = 0.05 and the confidence interval = 10%. Based on the estimation of the minimum sample size required, the sample sizes of the CAG group and CNAG group were equal, requiring 93 samples for both the CAG group and the CNAG group.

### Study design and participants

We performed a prospective nested case–control study. Our cohort consisted of 1539 patients who were at least 18 years old and volunteered to participate in this study to undergo gastroscopy in the digestive endoscopy center of our hospital from December 1, 2020 to July 1, 2021. This study protocol (XWKD-2020086) was approved by the ethics committee of Xuanwu Hospital of Capital Medical University. The written informed consent was obtained from all the participants in the study.

The endoscopist diagnosis procedure: In accordance with the guidelines [4], the endoscopist routinely took 3 biopsies from the gastric antrum, gastric angle and gastric body for each patient during the process of gastroscopy operation; additionally, another biopsy was taken from the suspected atrophy site. Olympus GIF-HQ290 was used to perform gastroscopy for patients, and Boston Scientific Radial Jaw 4 biopsy forceps were used to take biopsies.

The real-time video monitoring model for endoscopic diagnosis of CAG based on U-Net DL diagnosis procedure: Synchronized with the doctor's observations, the DL model also marked the suspected atrophy sites during real-time video monitoring of the same patient, after which the assistant informed the doctor to proceed with the biopsy of the suspected atrophy sites as labeled by the DL model. If the suspected atrophy site labeled by the DL model overlapped with the suspected atrophy site observed by the endoscopist, there was no need for another biopsy.

Based on the pathological results of the biopsy tissue, the patients in the cohort were divided into either the CAG group or the CNAG group, and the diagnostic evaluation indices of this model for the endoscopic diagnosis of CAG and its consistency with pathological diagnosis were evaluated.

The exclusion criteria were as follows: (1) Patients who could not tolerate gastroscopy and did not complete the procedure; (2) Patients who were found during gastroscopy to have lesions other than chronic gastritis, such as peptic ulcers or gastrointestinal malignancies; (3) Patients with contraindications to biopsy, such as taking anticoagulant or anti-platelet drugs; and (4) Patients who requested withdrawal from the study during gastroscopy.

### Diagnosis of chronic atrophic gastritis

All of our operations were carried out by endoscopists who had performed more than 10,000 gastroscopy procedures, who were experienced and who held the title of associate chief physician or above. According to guidelines [4], a pathological biopsy of chronic gastritis showing atrophy of the inherent glands could lead to a diagnosis of atrophic gastritis, regardless of the number of areas or degree of atrophy of the biopsy specimen. Biopsy histopathology is very important for the diagnosis of CAG, and biopsy should be performed according to the pathological conditions and needs. For clinical diagnosis, it is recommended to take 3 pieces of tissue for biopsy in gastric antrum, gastric angle and great curvature of the middle part of gastric body. Another biopsy was taken on the suspected lesions. Specimens should be large enough to reach the mucosal muscularis [15]. The severity of atrophy was estimated by the amount of natural glands reduced in the stomach (mild: the number of natural glands is reduced, not more than 1/3 of the original glands; moderate: the number of natural glands decreased between 1/3 and 2/3 of the original glands; severe: the number of natural glands is reduced by more than 2/3 of the original glands, with only a few remaining glands or even complete disappearance). The severity of CAG can be divided into mild, moderate and severe according to the pathological conditions or C type and O type according to the range of lesions [16]. The best noninvasive method to assess HP is the urea breath test (C13) with positive DOB ≥ 4.

### The real-time video monitoring model for endoscopic diagnosis of CAG based on U-Net deep learning

With the rapid development of DL technology, the application of DL in the field of medical imaging has attracted extensive research and attention, of which determining how to automatically identify and segment lesions in medical images is one of the most concerning problems. In order to solve this problem, the U-Net network model has been proposed [17, 18]. It is based on an FCN (fully convolutional network) and consists of an encoder, bottleneck module and decoder. Due to its U-shaped structure that combines context information, fast training speed and small amounts of data, it can meet the demands of medical image segmentation [19]. The classical DL model of image recognition requires a large amount of training data. Given that it is difficult for medical images to obtain such large-scale data, U-Net simply makes up for this deficiency. The main idea of U-Net is to add a network similar to the previous one behind the contracted network, in which the pooling operator is replaced by the upsampling operator. Therefore, these layers increase output resolution [20]. For localization, high-resolution features from the contraction path are combined with the upsampled output. The continuous convolutional layer can then learn to assemble a more accurate output based on this information. Since being proposed, the U-Net network has been widely used in medical image segmentation. U-Net has become the baseline model for most semantic segmentation tasks of medical images [21, 22]. This study intends to build a deep learning-based endoscopic diagnosis model for CAG by applying U-Net.

The INPAINT_TELEA algorithm is used to process watermarks in certain areas of the gastroscopic image, such as age, gender, time and system. The objective is to remove sensitive information related to patients and avoid white watermark interference in atrophic gastritis image recognition.

This model evaluates and measures the performance of the model through dice similarity coefficient and intersection over union (IOU), commonly used evaluation indexes for medical image segmentation. Dice and IOU are both measures to measure the similarity between two sets, and are used to measure the similarity between network segmentation results and standard masks in the field of image segmentation.

The cross entropy loss function is used in the loss function, Adam optimizer is used, the initial learning rate is 0.01, attenuation rate is 0.00003. The training hardware platform is a single-card server. The CPU is Intel Xeon (Cascade Lake) Platinum 8269 2.5 GHz, and the GPU is NVIDIA A100.

Our model analyzes images in real time and automatically during gastroscopy. Fully and accurately extract and store clear images of all detected parts and atrophic lesions from the global video, and arrange them according to the operation sequence of international standard [23]. Each atrophy lesion in each patient was automatically labeled and the atrophy severity was assessed. After the examination, the diagnosis of CAG on the patient level was made according to the guidelines [4].

### Deep learning model training and testing

In this study, a U-Net network was used to build a real-time video monitoring endoscopic diagnosis model for CAG based on DL.

This is done in three steps. The first is the preparation of the dataset: Based on the pathological diagnosis, 5290 high-quality endoscopic images of 1711 patients who underwent gastroscopy in our hospital from August 1, 2019 to August 1, 2020 were labeled by two gastroenterologists who had the experience of having performed more than 10,000 gastroscopy cases and who held the title of associate chief physician or above. A total of 4175 images of CAG were labeled, including 2389 images of mild atrophic gastritis, 977 images of moderate atrophic gastritis and 809 images of severe atrophic gastritis. In addition, 1115 images of CNAG were labeled. Then, according to the severity of atrophy, 70% of the images were included in the training set, and 30% of the images were included in the test set by stratified random method. The accuracy of the model was adjusted by fivefold cross validation with 3703 gastroscopy images.

The second step was the definition of the model: the concrete structure of the model definition. The left part was an encoder, which consisted of two 3 × 3 convolution layers (ReLU) and a 2 × 2 maxpooling layer to form a subsampling module. A total of four subsampling modules were connected together to form an encoder, and the encoder was then connected to the decoder in the right half. The decoder was repeatedly composed of a deconvolution layer + feature splicing concat + two 3 × 3 convolution layers (ReLU).

The third step was the training of the model. The training process involved taking a test image as the input of the U-Net model and obtaining the output after the model processing. The output results were compared with the results of labeled gastritis lesions, and a current loss value was calculated according to the loss function. The loss propagated backward along the network structure, the gradient of the parameters of this layer was calculated at each layer of the network, and the parameters were updated according to the gradient. The loss function here is the BCEWithLogitsLoss function, and the algorithm of parameter update adopts the adaptive optimization algorithm RMSProp. The whole dataset was divided into several batches, and the above process was repeated for each BATCH to update the model parameters until convergence. After all batches of training were completed, the new model parameters fit the characteristics of the training data well and were suitable for the diagnostic task of CAG.

After the model training was completed, we tested the model using 1587 endoscopic images. The sensitivity, specificity and accuracy of the model for the endoscopic diagnosis of CAG were 92.73%, 92.24% and 92.63%, respectively.

### Outcomes

Our primary outcome was to conduct a nested case–control study and to use the pathological diagnosis as the gold standard to study the sensitivity, specificity, accuracy and other diagnostic evaluation indices of the real-time video monitoring model for endoscopic diagnosis of CAG based on U-Net DL, evaluate its consistency with pathological diagnosis, and draw its receiver operating characteristic (ROC) curve.

Our secondary outcome was to use the pathological diagnosis as the gold standard to conduct subgroup analysis to evaluate the sensitivity, specificity, accuracy and other diagnostic evaluation indices of the real-time video monitoring model for endoscopic diagnosis of CAG based on U-Net DL in the diagnosis of mild, moderate and severe CAG.

### Statistical analysis

We assessed the diagnostic evaluation indices of the DL model after propensity score matching (PSM) to minimize the selection bias in this real-world study (RWS).

Given the differences in the baseline characteristics between eligible participants in the two groups (Table 1), PSM was used to identify a cohort of patients with similar baseline characteristics. The propensity score is a conditional probability of having a particular case–control (CAG vs. CNAG) given a set of baseline measured covariates [24]. The propensity score was estimated with the use of a nonparsimonious multivariate logistic regression model, with CAG as the dependent variable and all the baseline characteristics outlined in Table 1 as covariates. Matching was performed with the use of a 1:1 matching protocol without replacement (nearest-matching algorithm), with a caliper width equal to 0.2 of the standard deviation of the logit of the propensity score [25]. Standardized differences were estimated for all the baseline covariates before and after matching to assess prematch imbalance and postmatch balance. Standardized differences of less than 0.1 for a given covariate indicate a relatively small imbalance [26].

**Table 1:** Baseline characteristics before and after propensity score matching

Characteristic	Before matching			After matching		
	CAG (n = 338) (%)	CNAG (n = 793) (%)	Standardized difference	CAG (n = 338) (%)	CNAG (n = 338) (%)	Standardized difference
Sex (%)			− 0.0875			− 0.0266
Male	70.4	61.7		70.4	67.8	
Female	29.6	38.3		29.6	32.2	
Age			0.0158			0.0030
Distribution (%)						
< 40 yrs	8.9	10.6		8.9	9.8	
40–59 yrs	48.8	46.4		48.8	45.3	
60–75 yrs	33.4	34.7		33.4	38.2	
> 75 yrs	8.9	8.3		8.9	6.8	
Indication (%)			− 0.0370			0.0030
Screening	37.9	34.2		37.9	38.2	
Diagnosis	62.1	65.8		62.1	61.8	
HP (%)			− 0.0149			0.0059
Yes	26.6	28.1		26.6	26.0	
No	73.4	71.9		73.4	74.0	
Smoking (%)			0.0294			− 0.0207
Yes	31.1	28.1		31.1	33.1	
No	68.9	71.9		68.9	66.9	
Drinking (%)			− 0.0480			− 0.0030
Yes	21.3	26.1		21.3	21.6	
No	78.7	73.9		78.7	78.4	
HT (%)			− 0.0150			0.0059
Yes	32.5	34.0		32.5	32.0	
No	67.5	66.0		67.5	68.0	
CHD (%)			− 0.0326			0.0059
Yes	25.7	29.0		25.7	25.1	
No	74.3	71.0		74.3	74.9	
Diabetes (%)			0.0026			0.0030
Yes	24.9	24.6		24.9	24.5	
No	75.1	75.4		75.1	75.5	

*CAG* chronic atrophic gastritis, *CNAG* chronic nonatrophic gastritis, *HP* helicobacter pylori, *HT* hypertension, *CHD* coronary heart disease

Continuous variables are expressed as the mean and standard deviation (SD) or median and interquartile range (IQR) for skewed data, and categorical variables are expressed as frequencies (%). Continuous variables were compared using the t-test if normally distributed and the Mann–Whitney U test if not. Categorical variables were compared using the chi-square test or Fisher’s exact test. Using the data for the propensity-matched patients, ROC curves were constructed to assess sensitivity, specificity and respective areas under the curves (AUCs) with 95% CIs.

A two-tailed *P* value < 0.05 was considered statistically significant. All of the analyses were conducted using SPSS software, version 23.0 (IBM Corp., Armonk, NY, USA).

### Sensitivity analysis

To test the robustness of the main results, several additional analyses were conducted. First, using the data for all the patients before matching, we assessed the diagnostic evaluation indices of the DL model. Second, subgroup analysis with the data before matching was also conducted by stratifying CAG patients into mild, moderate or severe groups.

## Results

### Study population

**Fig. 1:**
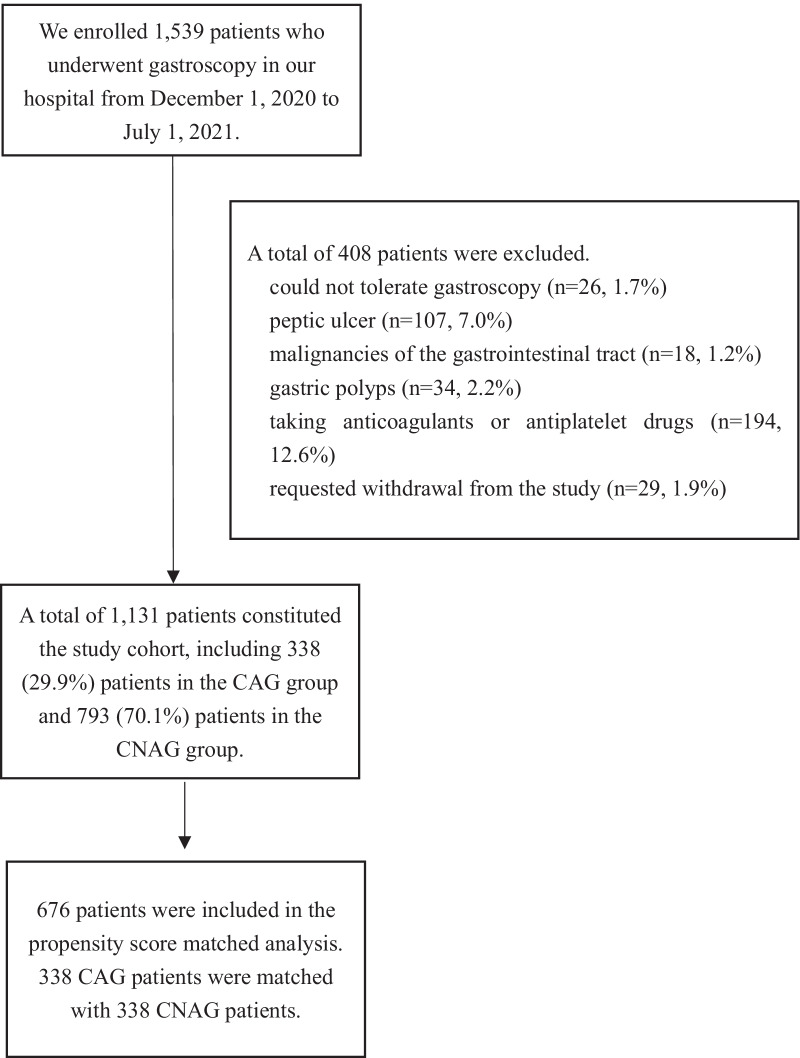
Flow chart of the identification of the study sample

Figure 1 shows the study flowchart. A total of 1539 patients who underwent gastroscopy in the digestive endoscopy center of our hospital were enrolled in the study. A total of 408 patients were excluded. Reasons for exclusion included: Patients who were unable to tolerate gastroscopy and did not complete the procedure (n = 26, 1.7%); patients with peptic ulcer disease found during gastroscopy (n = 107, 7.0%); patients with gastrointestinal malignancies found during gastroscopy (n = 18, 1.2%); patients with gastric polyps found during gastroscopy (n = 34, 2.2%); patients with contraindications to biopsy, such as taking anticoagulant or antiplatelet drugs (n = 194, 12.6%); and patients who requested withdrawal from the study during gastroscopy (n = 29, 1.9%).

A total of 1131 patients constituted the study cohort, including 338 (29.9%) patients in the CAG group and 793 (70.1%) patients in the CNAG group. Before PSM, there were differences between the two groups in several of the baseline variables (Table 1). With the use of PSM, 338 CAG patients were matched with 338 CNAG patients. After matching, the standardized differences were less than 0.1 for all variables, indicating only small differences between the two groups (Table 1).

### Primary outcomes

**Fig. 2:**
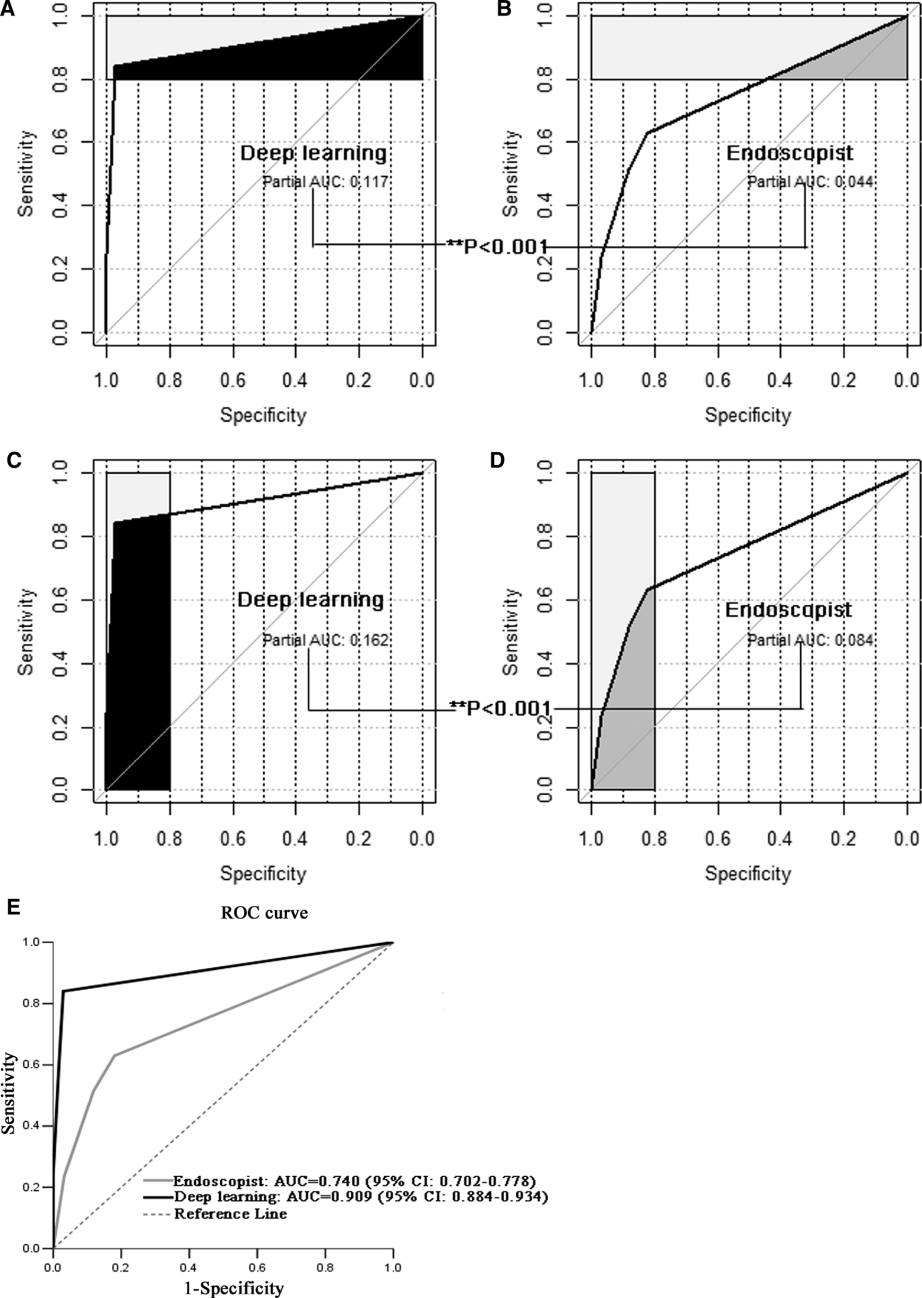
The diagnostic performance comparison between DL group and endoscopist group when taking pathological diagnosis as the gold standard. **A** Partial AUC (The black shaded part) at the sensitivity ≥ 0.8 for DL group. **B** Partial AUC (The dark grey shaded part) at the sensitivity ≥ 0.8 for endoscopist group. **C** Partial AUC ((The black shaded part) at the specificity ≥ 0.8 for DL group. **D** Partial AUC (The dark grey shaded part) at the specificity ≥ 0.8 for endoscopist group. **E** ROC curves for DL group and endoscopist group respectively. After matching and taking pathological diagnosis as the gold standard, the diagnostic evaluation indices and the evaluation of consistency with pathological diagnosis in the DL group were better than those in the endoscopist group

We conducted a nested case–control study with the present cohort. After matching and taking pathological diagnosis as the gold standard, the diagnostic evaluation indices and consistency evaluation of the real-time video monitoring model for endoscopic diagnosis of CAG based on U-Net DL were better than those of endoscopists (Table 2, Fig. 2).

**Table 2:** Diagnostic evaluation indices and the evaluation of consistency with pathological diagnosis in the deep learning group and endoscopist group before and after propensity score matching

CAG versus CNAG	Before matching (338 vs. 793)		After matching (338 vs. 338)	
	DL	Endoscopist	DL	Endoscopist
Sensitivity	84.02%	62.72%	84.02%	62.72%
Specificity	96.34%	80.45%	97.04%	81.95%
PV+	90.73%	57.77%	96.60%	77.66%
PV−	93.40%	83.51%	85.86%	68.73%
Accuracy	92.66%	75.15%	90.53%	72.34%
Youden index	80.36%	43.17%	81.06%	44.67%
Odd product	91.71	6.93	172.5	7.64
LR +	22.96	3.21	28.39	3.47
LR−	0.17	0.46	0.16	0.45
AUC (95% CI)	0.906 (0.882–0.930)	0.735 (0.700–0.769)	0.909 (0.884–0.934)	0.740 (0.702–0.778)
Kappa	0.842	0.492	0.852	0.558

*DL* deep learning, *PV*+ positive predictive value, *PV− *negative predictive value, *LR*+ positive likelihood ratio, *LR− *negative likelihood ratio

### Secondary outcomes

With pathological diagnosis as the gold standard, subgroup analysis was conducted. After matching, the sensitivity, specificity, accuracy and other diagnostic evaluation indices of the real-time video monitoring diagnosis model for CAG based on U-Net DL were better than those of endoscopists in the diagnosis of mild, moderate and severe CAG (Table 3).

**Table 3:** Diagnostic evaluation indices in the deep learning group and endoscopist group after propensity score matching in subgroups for the severity of CAG

	Mild CAG versus CNAG (104 vs. 338)		Moderate CAG versus CNAG (147 vs. 338)		Severe CAG versus CNAG (87 vs. 338)	
	DL	Endoscopist	DL	Endoscopist	DL	Endoscopist
Sensitivity	72.12%	39.42%	85.71%	62.59%	95.40%	90.80%
Specificity	97.04%	81.95%	97.04%	81.95%	97.04%	81.95%
PV+	88.34%	40.20%	92.65%	60.13%	89.25%	56.43%
PV−	91.88%	81.47%	93.98%	83.43%	98.80%	97.19%
Accuracy	91.18%	71.95%	93.61%	76.08%	96.71%	83.76%
Youden index	69.16%	21.37%	82.75%	44.54%	92.44%	72.75%
Odd product	84.83	2.96	196.8	7.6	680.6	44.84
LR+	24.36	2.18	28.96	3.47	32.23	5.03
LR−	0.29	0.74	0.15	0.46	0.05	0.11

*DL* deep learning, *PV*+ positive predictive value, *PV− *negative predictive value, *LR*+ positive likelihood ratio, *LR− *negative likelihood ratio

### Sensitivity analysis

We performed statistical analysis on all patients before matching and obtained similar results (Table 2).

Subgroup analysis with the data before matching was also conducted by stratifying CAG patients into the mild, moderate or severe groups. With pathological diagnosis as the gold standard, in all subgroup analyses before matching, the sensitivity, specificity, accuracy and other diagnostic evaluation indices of the real-time video monitoring model for endoscopic diagnosis of CAG based on U-Net DL were better than those of endoscopists in the diagnosis of mild, moderate and severe CAG (Table 4).

**Table 4:** Diagnostic evaluation indices in the deep learning group and endoscopist group before propensity score matching in subgroups for the severity of CAG

	Mild CAG versus CNAG (104 vs. 793)		Moderate CAG versus CNAG (147 vs. 793)		Severe CAG versus CNAG (87 vs. 793)	
	DL	Endoscopist	DL	Endoscopist	DL	Endoscopist
Sensitivity	72.12%	39.42%	85.71%	62.59%	95.40%	90.80%
Specificity	96.34%	80.45%	96.34%	80.45%	96.34%	80.45%
PV+	72.12%	20.92%	81.29%	37.25%	74.11%	33.76%
PV−	96.34%	91.01%	97.32%	92.06%	99.48%	98.76%
Accuracy	93.53%	75.70%	94.68%	77.66%	96.25%	81.48%
Youden index	68.46%	19.87%	82.05%	43.04%	91.74%	71.25%
Odd product	68.13	2.68	158.07	6.89	546.66	40.65
LR+	19.70	2.02	23.42	3.2	26.07	4.64
LR−	0.29	0.75	0.15	0.47	0.05	0.11

*DL* deep learning, *PV*+ positive predictive value, *PV− *negative predictive value, *LR*+ positive likelihood ratio, *LR− *negative likelihood ratio

## Discussion

The morbidity and mortality of GC in China rank first in the world. Reducing the morbidity and mortality of GC in China is one of the major public health problems that urgently need to be solved [1]. A prospective study of 1592 patients with CAG by Chinese scholars revealed the progression of CAG. Among them, 23 patients (1.44%) had GC due to CAG, and 349 patients (21.92%) had atypical hyperplasia. As age increased, atrophy and intestinal metaplasia deteriorated in more than 35% of patients [27]. Early detection and diagnosis of CAG can prevent the formation of GC to a certain extent, but the difficulty of diagnosis and the rate of missed diagnoses have brought great challenges to endoscopists [28]. According to the "Consensus of Chronic Gastritis in China", the endoscopic manifestations of CAG are red and white mucosa, mainly white mucosa, folds that flatten or even disappear, and exposure of some mucosal vessels. These features may be accompanied by mucosal granules or nodules [4]. However, in clinical practice, identifying mucosal atrophy is mainly based on the subjective impression of endoscopists and depends on their understanding of the guidelines, previous operating experience, and the standard training level conducted by the hospital and other factors. Therefore, the diagnosis of CAG solely dependent on endoscopists is uncertain and varies greatly [29]. Studies have shown that the proportion of endoscopic diagnoses for CAG varies greatly in different regions and in different hospitals in the same region, fluctuating from 17.7 to 39.8%, and the sensitivity of the endoscopic diagnosis of CAG is only 42% [28]. Endoscopic atrophy classification exhibited a significant correlation between histological atrophy and intestinal metaplasia, and represents a noninvasive classification method [16, 30]. Endoscopic grading can predict histological atrophy with few false negatives, indicating that precancerous conditions can be identified during screening endoscopy [31, 32]. Therefore, it is particularly important to improve the endoscopic diagnosis rate of CAG. Determining how to achieve consistent and accurate early detection and diagnosis of CAG by every endoscopist has always been a difficult problem that clinical guidelines have been trying but have been unable to solve.

DL is an improvement of artificial neural networks, which are composed of more layers of neural networks, allowing the higher layer to contain more abstract information for data prediction. To date, DL has become the leading machine learning tool in the field of computer vision [5, 19, 33]. A typical convolutional neural network (CNN) model used for image processing in DL consists of a series of convolutional networks, including a series of convolutional layers, pooling layers and fully connected layers. Like low-level visual processing in the human brain, convolutional network detection extracts image features, such as lines or circles that might represent straight edges (such as organ detection) or circles (colon polyp detection), followed by higher-order features, such as local and global shape or texture feature extraction [6, 34]. CNN needs to acquire a large amount of training data, while medical images have difficulty obtaining such large-scale data [11]. Therefore, a kind of network model, namely U-Net, which is especially suitable for biomedical image processing tasks, is emerging at the right moment. The main idea of U-Net is to supplement a network similar to the previous one after the contraction network, in which the pooling operator is replaced by the upsampling operator. Therefore, these layers increase the resolution of the output. For localization, the high-resolution features from the contraction path are combined with the upsampled output. The continuous convolutional layer can then learn to assemble a more accurate output based on this information [35–37]. Since being proposed, the U-Net network has been widely used in medical image segmentation. U-Net was first published in MICCAI in 2015 and then became the baseline model for most of the semantic segmentation tasks of medical images [38]. It also inspired a large number of researchers to think about U-shaped semantic segmentation networks. In the field of natural image understanding, an increasing number of semantic segmentation and target detection SOTA models have begun to pay attention to and use U-shaped structures [39–41].

The application of DL combined with digestive endoscopy has become a research hot topic, especially for the diagnosis of upper digestive tract diseases [42]. At present, the main research directions focus on DL auxiliary detection of Barrett's esophagus, auxiliary detection of esophageal cancer, auxiliary detection of GC, auxiliary detection of *Helicobacter pylori* infection and auxiliary identification of anatomical sites, especially for early cancer [43]. Some scholars have applied AI to the study of traditional endoscopy. After machine learning through upper digestive tract endoscopic images, the sensitivity of malignant lesions was as high as 98%, and the negative predictive value was 95%, but the positive predictive value was only 40%. It is possible to improve the positive predictive value by increasing the number of learning samples [8]. By using a large number of traditional endoscopic images and using the convolutional neural network in the DL algorithm, some scholars have established a computer-aided diagnosis (CAD) system capable of automatic detection of early GC. It can identify lesions quickly and has a sensitivity of 92%, indicating that the CAD system with this algorithm as the core has strong clinical diagnostic ability [44]. While many scholars focus on early cancer of the upper digestive tract, our study focuses on early lesions of "early gastric cancer", "chronic atrophic gastritis", so as to "move forward the threshold" and more effectively reduce the occurrence of GC. Studies have shown that the accuracy, sensitivity and specificity of the convolutional neural network model for the diagnosis of atrophic gastritis are 0.942, 0.945 and 0.940, respectively, which are all higher than those of ordinary endoscopic experts, while the detection rates of mild, moderate and severe atrophic gastritis are 93%, 95% and 99%, respectively [12, 29]. However, the data used for the training and validation of the model in the above studies were all retrospective endoscopic static images, and the data were artificially preliminarily screened, thus lacking prospective research results. At present, prospective studies mainly focus on the recognition of static images, while the recognition of real-time surveillance video is limited. Our study extends the above studies well, develops a U-Net DL model for the diagnosis of CAG that can be applied in real-time video monitoring of gastroscopy, and conducts a prospective nested case–control study using PSM.

In our study, pathological diagnosis was taken as the gold standard, and it was found that the diagnostic evaluation indices and consistency evaluation of the real-time video monitoring model for endoscopic diagnosis of CAG based on U-Net DL were better than those of endoscopists. The sensitivity (84.02% vs. 62.72%) and specificity (97.04% vs. 81.95%) showed that the model had good ability to detect CAG and identify CNAG. The positive predictive value (96.60% vs. 77.66%) and negative predictive value (85.86% vs. 68.73%) showed that the patients with a positive diagnosis were more likely to be diagnosed with CAG, and the patients with a negative diagnosis were more likely to be diagnosed with CNAG. The accuracy rate (90.53% vs. 72.34%) showed that the diagnostic ability of CAG and CNAG was good. The Youden index (81.06% vs. 44.67%) showed that the model was more authentic. The odd product (172.5 vs. 7.64) showed that the diagnostic value of this model was high. The positive likelihood ratio (28.39 vs. 3.47) and negative likelihood ratio (0.16 vs. 0.45) indicated that the model had a good ability to detect CAG and identify CNAG when excluding the influence of prevalence; AUC (95% CI) [0.909 (0.884–0.934) vs. 0.740 (0.702–0.778)] and Kappa (0.852 vs. 0.558). The AUC of this model was > 0.9, indicating a high diagnostic accuracy. The Kappa of this model was > 0.8, indicating that it has better consistency with pathological diagnosis. Accurate diagnosis of CAG has always been difficult in gastroscopy, and the sensitivity of endoscopists is only 42%. The basic reason for the low sensitivity and accuracy of endoscopists in the diagnosis of CAG is that the diagnosis is mainly made through the subjective observation of gastric mucosal morphological characteristics under gastroscopy and the lack of quantitative indicators. At the same time, only the morphological description of CAG is given in the guidelines, and there is no quantitative standard. Therefore, the subjective judgment of endoscopists is likely to lead to misdiagnosis and missed diagnoses, and even the same doctor may draw different conclusions when observing the same case at different times. Our model well makes up for the above deficiencies. As a physician's assistant, it can objectively, stably and efficiently diagnose CAG.

At the same time, we conducted a subgroup analysis, and after matching, the sensitivity, specificity, accuracy and other diagnostic evaluation indices of the endoscopic diagnosis model for CAG based on U-Net DL were better than those of endoscopists in the diagnosis of mild, moderate and severe CAG. The sensitivity (72.12% vs. 39.42%, 85.71% vs. 62.59%, 95.40% vs. 90.80%, respectively) and the specificity (97.04% vs. 81.95% for both subgroups) of the model in the diagnosis of mild, moderate and severe CAG showed that its ability to detect mild, moderate and severe CAG was superior to that of endoscopists. The positive predictive values (88.34% vs. 40.20%, 92.65% vs. 60.13%, 89.25% vs. 56.43%, respectively) and the negative predictive values (91.88% vs. 81.47%, 93.98% vs. 83.43%, 98.80% vs. 97.19%, respectively) of the model indicated that patients diagnosed with mild, moderate and severe CAG had a higher probability of being diagnosed with mild, moderate and severe CAG compared to those found by endoscopists, while patients diagnosed with CNAG had a higher probability of being diagnosed with CNAG compared to that found by endoscopists. The accuracy rate (91.18% vs. 71.95%, 93.61% vs. 76.08%, 96.71% vs. 83.76%, respectively) of the model showed that its ability to diagnose mild, moderate and severe CAG and CNAG was better than that of endoscopists. The Youden index (69.16% vs. 21.37%, 82.75% vs. 44.54%, 92.44% vs. 72.75%, respectively) showed that this model for the diagnosis of mild, moderate and severe CAG was more authentic than that of endoscopists. The odds product (84.83 vs. 2.96, 196.8 vs. 7.6, 680.6 vs. 44.84, respectively) showed that the model was more valuable than that of endoscopists in the diagnosis of mild, moderate and severe CAG. The positive likelihood ratio (24.36 vs. 2.18, 28.96 vs. 3.47, 32.23 vs. 5.03, respectively) and the negative likelihood ratio (0.29 vs. 0.74, 0.15 vs. 0.46, 0.05 vs. 0.11, respectively) showed that, when the influence of prevalence was excluded, the ability of this model to detect mild, moderate and severe CAG and to identify CNAG was better than that of endoscopists. The consensus points out that moderate to severe CAG has a certain cancer rate, and the operative link for the gastritis assessment (OLGA) system based on the severity of CAG is an important system for the clinical assessment of the morbidity of GC. Studies have shown that the gastritis stage remains unchanged in the vast majority of OLGA 0-II patients, whereas cancer occurs in OLGA III and IV patients. An OLGA stage of high-risk grade III or IV is closely related to a high risk of GC, but the consistency rate of judgment between endoscopists and pathological diagnosis is relatively low [45]. Our model solves the above problems well and can assist endoscopists in accurately judging the severity of CAG, so as to avoid missed diagnoses in high-risk populations and effectively prevent the occurrence of GC.

Our study had some limitations. First, as this is an exploratory study, we conducted a nested case–control study with a cohort from our single-center. The enrolled cases were all from our region, which may have selection bias. In the near future, we will include cases from different regions for a multi-center study to make our results more representative. Second, in order to avoid risk to patients and improve the accuracy of the model, our exclusion criteria were relatively strict, excluding patients with lesions other than chronic gastritis, such as peptic ulcers and gastrointestinal malignant tumors, found during gastroscopy. Therefore, there was a certain bias in the patients enrolled in the cohort. Given the successful experience of the present study, our cohort will be included in a wider range of patients from multiple centers and will enroll patients who have chronic gastritis that is complicated by other lesions in subsequent studies, so as to more scientifically verify the effectiveness of our model. Third, Serological tests were not included in our analysis. The combination of pepsinogen I to pepsinogen II ratio (PGR), HP antibody and gastrin 17 has been shown to screen for gastric mucosal atrophy and is referred to as a "serological biopsy" [46, 47]. The combination of non-invasive serological screening and endoscopy can improve the screening effect of gastric cancer [48]. Our follow-up study will combine our model with “serological biopsy” results for statistical analysis to make our model more reliable.

## Conclusion

In conclusion, our prospective nested case–control study proves that, when taking pathological diagnosis as the gold standard, the diagnostic evaluation indices and consistency evaluation of the real-time video monitoring model for endoscopic diagnosis of CAG based on U-Net DL were superior to those of endoscopists and can better assist endoscopists in the real-time endoscopic diagnosis of CAG.

## Data Availability

The dataset generated and analyzed during the study is stored in a secure localized database but is available from the corresponding author in an anonymous format on reasonable request.

## Abbreviations

CAG: Chronic atrophic gastritis
GC: Gastric cancer
DL: Deep learning
CNAG: Chronic nonatrophic gastritis
PSM: Propensity score matching
AI: Artificial intelligence
ROC: Receiver operating characteristic
CAD: Computer-aided diagnosis

## References

[1] Zhuan L, Tao S, Hao W, Fan Y, Wenbing Z (2014). Consensus on early gastric cancer screening and endoscopic diagnosis and treatment in China (2014, Changsha). Chin J Digest.

[2] Uemura N, Okamoto S, Yamamoto S, Matsumura N, Yamaguchi S, Yamakido M (2001). *Helicobacter pylori* infection and the development of gastric cancer. N Engl J Med.

[3] Masuyama H, Yoshitake N, Sasai T, Nakamura T, Masuyama A, Zuiki T (2015). Relationship between the degree of endoscopic atrophy of the gastric mucosa and carcinogenic risk. Digestion.

[4] Jingyuan F, Du Y, Wenzhong L, Jianlin R, Yanqing L, Xiaoyu C (2017). Consensus on chronic gastritis in China (2017, Shanghai). Chin J Digest.

[5] Chen L, Papandreou G, Kokkinos I, Murphy K, Yuille AL (2018). DeepLab: semantic image segmentation with deep convolutional nets, atrous convolution, and fully connected CRFs. IEEE Trans Pattern Anal.

[6] Litjens G, Kooi T, Bejnordi BE, Setio AAA, Ciompi F, Ghafoorian M (2017). A survey on deep learning in medical image analysis. Med Image Anal.

[7] Kuwahara T, Hara K, Mizuno N, Haba S, Okuno N, Kuraishi Y (2022). Artificial intelligence using deep learning analysis of endoscopic ultrasonography images for the differential diagnosis of pancreatic masses. Endoscopy.

[8] Horie Y, Yoshio T, Aoyama K, Yoshimizu S, Horiuchi Y, Ishiyama A (2019). Diagnostic outcomes of esophageal cancer by artificial intelligence using convolutional neural networks. Gastrointest Endosc.

[9] Li L, Chen Y, Shen Z, Zhang X, Sang J, Ding Y (2020). Convolutional neural network for the diagnosis of early gastric cancer based on magnifying narrow band imaging. Gastric Cancer Off J Int Gastric Cancer Assoc Jpn Gastric Cancer Assoc.

[10] Higuchi N, Hiraga H, Sasaki Y, Hiraga N, Igarashi S, Hasui K (2022). Automated evaluation of colon capsule endoscopic severity of ulcerative colitis using ResNet50. PLoS ONE.

[11] Mori Y, Kudo SE, Mohmed HEN, Misawa M, Ogata N, Itoh H (2019). Artificial intelligence and upper gastrointestinal endoscopy: current status and future perspective. Digest Endosc.

[12] Guimarães P, Keller A, Fehlmann T, Lammert F, Casper M (2019). Deep-learning based detection of gastric precancerous conditions. Gut.

[13] Zhang X, Hu W, Chen F, Liu J, Yang Y, Wang L (2017). Gastric precancerous diseases classification using CNN with a concise model. PLoS ONE.

[14] Zhao Q, Chi T (2022). Deep learning model can improve the diagnosis rate of endoscopic chronic atrophic gastritis: a prospective cohort study. BMC Gastroenterol.

[15] Tytgat GNJ (1991). The Sydney System: endoscopic division. Endoscopic appearances in gastritis/duodenitis. J Gastroenterol Hepatol.

[16] Kimura K, Takemoto T (1969). An endoscopic recognition of the atrophic border and its significance in chronic gastritis. Endoscopy.

[17] Cao Y, Vassantachart A, Ye JC, Yu C, Ruan D, Sheng K (2021). Automatic detection and segmentation of multiple brain metastases on magnetic resonance image using asymmetric UNet architecture. Phys Med Biol.

[18] Zhou Z, Siddiquee MMR, Tajbakhsh N, Liang J (2020). UNet++: redesigning skip connections to exploit multiscale features in image segmentation. IEEE Trans Med Imaging.

[19] Tang P, Liang Q, Yan X, Xiang S, Sun W, Zhang D (2019). Efficient skin lesion segmentation using separable-Unet with stochastic weight averaging. Comput Meth Prog Biomed.

[20] Jin Q, Meng Z, Sun C, Cui H, Su R (2020). RA-UNet: a hybrid deep attention-aware network to extract liver and tumor in CT scans. Front Bioeng Biotechnol.

[21] Li J, Lin X, Che H, Li H, Qian X (2021). Pancreas segmentation with probabilistic map guided bi-directional recurrent UNet. Phys Med Biol.

[22] Zhao B, Zhang X, Li Z, Hu X (2019). A multi-scale strategy for deep semantic segmentation with convolutional neural networks. Neurocomputing.

[23] Hirasawa T, Uchita K, Yano T (2016). How many pictures are demanded for screening gastroscopy?. Digest Endosc.

[24] Rosenbaum PR, Rubin DB (1983). The central role of the propensity score in observational studies for causal effects. Biometrika.

[25] Zhao Q, Chi T (2021). Biopsy in emergency gastroscopy does not increase the risk of rebleeding in patients with Forrest I acute nonvariceal upper gastrointestinal bleeding combined with suspected malignant gastric ulcer: a multicenter retrospective cohort study. BMC Gastroenterol.

[26] Rubin DB, Rubin DB (2006). Using propensity scores to help design observational studies: application to the tobacco litigation. Health Serv Outcomes Res Methodol.

[27] Chooi EY, Chen HM, Miao Q, Weng YR, Chen XY, Ge ZZ (2012). Chronic atrophic gastritis is a progressive disease: analysis of medical reports from Shanghai (1985–2009). Singapore Med J.

[28] Junxiang L, Yan C, Bin L, Yangang W (2018). Consensus on the diagnosis and treatment of chronic atrophic gastritis with integrated traditional chinese and western medicine (2017). Chin J Integr Tradit Western Med Digest.

[29] Zhang Y, Li F, Yuan F, Zhang K, Huo L, Dong Z (2020). Diagnosing chronic atrophic gastritis by gastroscopy using artificial intelligence. Digest Liver Dis.

[30] Kodama M, Okimoto T, Ogawa R, Mizukami K, Murakami K (2015). Endoscopic atrophic classification before and after *H. pylori* eradication is closely associated with histological atrophy and intestinal metaplasia. Endosc Int Open.

[31] Kono S (2015). Can endoscopic atrophy predict histological atrophy? Historical study in United Kingdom and Japan. World J Gastroenterol.

[32] Sugano K, Tack J, Kuipers EJ, Graham DY, El-Omar EM, Miura S (2015). Kyoto global consensus report on *Helicobacter pylori* gastritis. Gut.

[33] Fitting D, Krenzer A, Troya J, Banck M, Sudarevic B, Brand M (2022). A video based benchmark data set (ENDOTEST) to evaluate computer-aided polyp detection systems. Scand J Gastroenterol.

[34] Glissen Brown JR, Mansour NM, Wang P, Chuchuca MA, Minchenberg SB, Chandnani M (2022). Deep learning computer-aided polyp detection reduces adenoma miss rate: a United States Multi-center Randomized Tandem Colonoscopy Study (CADeT-CS Trial). Clin Gastroenterol Hepatol.

[35] Li D, Chen C, Li J, Wang L (2020). Dense gate network for biomedical image segmentation. Int J Comput Assist Radiol.

[36] Chen Y, Wang K, Liao X, Qian Y, Wang Q, Yuan Z (2019). Channel-Unet: a spatial channel-wise convolutional neural network for liver and tumors segmentation. Front Genet.

[37] Thomas E, Pawan SJ, Kumar S, Horo A, Niyas S, Vinayagamani S (2021). Multi-Res-Attention UNet: a CNN model for the segmentation of focal cortical dysplasia lesions from magnetic resonance images. IEEE J Biomed Health.

[38] Zhang Y, Wu J, Liu Y, Chen Y, Wu EX, Tang X (2021). MI-UNet: multi-inputs UNet incorporating brain parcellation for stroke lesion segmentation from T1-weighted magnetic resonance images. IEEE J Biomed Health.

[39] Ibtehaz N, Rahman MS (2020). MultiResUNet: rethinking the U-Net architecture for multimodal biomedical image segmentation. Neural Netw.

[40] Moustafa MS, Mohamed SA, Ahmed S, Nasr AH (2021). Hyperspectral change detection based on modification of UNet neural networks. J Appl Remote Sens.

[41] Zhu C, Mei K, Peng T, Luo Y, Liu J, Wang Y (2021). Multi-level colonoscopy malignant tissue detection with adversarial CAC-UNet. Neurocomputing.

[42] Suqin L, Shan H, Yiyun C, Xiaoyu Z, Xia L, Honggang Y (2020). Advances in the application of artificial intelligence in digestive endoscopy. Chin J Digest Endosc.

[43] Shengbing Z, Wei Q, Yu B, Zhaoshen L (2019). Advances in the application of artificial intelligence in the diagnosis and treatment of digestive endoscopy. Chin J Digest Endosc.

[44] Hirasawa T, Aoyama K, Tanimoto T, Ishihara S, Shichijo S, Ozawa T (2018). Application of artificial intelligence using a convolutional neural network for detecting gastric cancer in endoscopic images. Gastric Cancer.

[45] Yue H, Shan L, Bin L (2018). The significance of OLGA and OLGIM staging systems in the risk assessment of gastric cancer: a systematic review and meta-analysis. Gastric Cancer.

[46] Agréus L, Kuipers EJ, Kupcinskas L, Malfertheiner P, Di Mario F, Leja M (2012). Rationale in diagnosis and screening of atrophic gastritis with stomach-specific plasma biomarkers. Scand J Gastroenterol.

[47] Michigami Y, Watari J, Ito C, Nakai K, Yamasaki T, Kondo T (2018). Long-term effects of *H. pylori* eradication on epigenetic alterations related to gastric carcinogenesis. Sci Rep UK.

[48] Tu H, Sun L, Dong X, Gong Y, Xu Q, Jing J (2017). A serological biopsy using five stomach-specific circulating biomarkers for gastric cancer risk assessment: a multi-phase study. Am J Gastroenterol.

